# Report from the 26th Annual Western Canadian Gastrointestinal Cancer Consensus Conference on Hepatocellular and Biliary Tract Cancer, Saskatoon, Saskatchewan, 17–18 October 2024

**DOI:** 10.3390/curroncol32070398

**Published:** 2025-07-10

**Authors:** Deepti Ravi, Shahid Ahmed, Blaire Anderson, Brady Anderson, Bryan Brunet, Haji Chalchal, Arun Elangovan, Georgia Geller, Vallerie Gordon, Branawan Gowrishankar, Edward Hardy, Mussawar Iqbal, Duc Le, Richard Lee-Ying, Shazia Mahmood, Karen Mulder, Maged Nashed, Killian Newman, Maurice Ogaick, Vibhay Pareek, Jennifer Rauw, Ralph Wong, Adnan Zaidi

**Affiliations:** 1 Saskatchewan Health Authority, Saskatoon, SK S7K 0M7, Canada; 2 Saskatoon Cancer Centre, Saskatoon, SK S7N 4H4, Canada; shahid.ahmed@saskcancer.ca (S.A.); bryan.brunet@saskcancer.ca (B.B.); arun.elangovan@saskcancer.ca (A.E.); vallerie.gordon@saskcancer.ca (V.G.); duc.le@saskcancer.ca (D.L.); adnan.zaidi@saskcancer.ca (A.Z.); 3 College of Medicine, University of Alberta, Edmonton, AB T6G 2B7, Canada; blaire.anderson@albertahealthservices.ca; 4 Western Manitoba Cancer Center, Brandon, MB R7A 5M8, Canada; banderson6@manitoba-physicians.ca; 5 Allan Blair Cancer Centre, Regina, SK S4T 7T1, Canada; haji.chalchal@saskcancer.ca (H.C.); mussawar.iqbal@saskcancer.ca (M.I.); shazia.mahmood@saskcancer.ca (S.M.); 6 BC Cancer Centre, Victoria, BC V8R 6V5, Canada; ggeller@bccancer.bc.ca; 7 Cross Cancer Institute, Alberta Health Services, Edmonton, AB T6G 1Z2, Canada; branawan.gowrishankar@albertahealthservices.ca (B.G.); karen.mulder@ahs.ca (K.M.); jennifer.rauw@albertahealthservices.ca (J.R.); 8 BC Cancer Centre, Vernon, BC V1T 5L2, Canada; ehardy@bccancer.bc.ca; 9 Tom Baker Cancer Centre, Calgary, AB T2N 4N2, Canada; richard.lee-ying@ahs.ca; 10 CancerCare Manitoba, Winnipeg, MB R3E 0V9, Canada; mnashed1@cancercare.mb.ca (M.N.); vpareek@cancercare.mb.ca (V.P.); rwong2@cancercare.mb.ca (R.W.); 11 Foothills Medical Centre, Calgary, AB T2N 2T9, Canada; killian.newman@ucalgary.ca; 12 College of Medicine, University of Saskatchewan, Saskatoon, SK S7N 5A2, Canada; maurice.ogaick@saskatoonhealthregion.ca

**Keywords:** adjuvant chemoradiation, adjuvant radiation therapy, adjuvant systemic therapy, biliary tract cancer, cholangiocarcinoma, extrahepatic cholangiocarcinoma, gallbladder adenocarcinoma, hepatectomy, hepatocellular carcinoma, HER2, intrahepatic cholangiocarcinoma, MMR, molecular profiling, systemic therapy

## Abstract

The 26th annual Western Canadian Gastrointestinal Cancer Consensus Conference (WCGCCC) was held in Saskatoon, Saskatchewan, on 17–18 October 2024. The WCGCCC is an interactive multidisciplinary conference that was attended by healthcare professionals from across Western Canada (British Columbia, Alberta, Saskatchewan, and Manitoba) who are involved in the care of patients with hepatocellular and biliary tract cancers. Specialists from the fields of medical and radiation oncology, interventional radiology, pathology and laboratory medicine, and general and hepatobiliary surgery participated in presentations and discussions for the purpose of developing the recommendations presented here. This consensus statement addresses current issues in the management of hepatocellular and biliary tract cancers.

## Simple Summary

This paper provides recommendations for healthcare professionals involved in the care of patients with hepatocellular and biliary tract carcinoma based on current published criteria. Patients should be assessed by a multi-disciplinary team. Surgically, achievement of functional liver remnant with negative margins is important for all resection cases. Patients should be counselled prior to molecular testing as mutations may not be actionable. Actionable mutations for biliary tract cancer include HER2, BRAF V600E, NTRK fusion, and RAS. DNA mismatch repair (MMR) is recommended for all patients. Combination therapy (bevacizumab and atezolizumab) or immunotherapy are preferred first-line options for advanced hepatocellular carcinoma (HCC). Further studies are required to prove if a combination of local and systemic therapy can be utilized widely in patients with intermediate-risk HCC. For biliary cancers, a combination of platinum, gemcitabine, and immunotherapy with durvalumab or pembrolizumab is the optimal systemic treatment, if available.

## 1. Terms of Reference

### 1.1. Purpose

The aim of the Western Canadian Gastrointestinal Cancer Consensus Conference (WCGCCC) is to establish clinical consensus by engaging specialists from across Western Canada, who are working in the fields of medical and radiation oncology, interventional radiology, pathology and laboratory medicine, and general and hepatobiliary surgery. By defining best clinical practice, the participants of the WCGCCC strive to improve the care and clinical outcomes for patients with hepatocellular and biliary tract cancers.

### 1.2. Participants

The WCGCCC welcomes health professionals from Western Canada who are involved in the care of patients with hepatocellular and biliary tract cancers (Table 1). Participants are provided the clinical questions to be addressed during the consensus conference in advance (Table 2).

### 1.3. Target Audience

The recommendations presented here are written for healthcare professionals involved in the care of patients with hepatocellular and biliary tract cancers.

### 1.4. Basis of Recommendations

The recommendations are based on presentation and discussion of the best available evidence. References are cited where applicable.

## 2. Question 1—What Are the Current Criteria for Curative Surgery and Transplant Candidacy in Patients with Hepatocellular Carcinoma?

### 2.1. Recommendations

- Patients should be assessed by a multidisciplinary team to review treatment options, ideally in a recurrent fashion. Representation of key specialties—ideally surgical, transplant, radiology, interventional radiology, hepatology, radiation and, medical oncology specialties—enriches rounds. Patients with liver-limited disease may be candidates for transplant and early involvement with transplant is advised.
- Achievement of adequate functional liver remnant and negative margins is key for all surgical resection cases. Resection may be considered for patients with compensated cirrhosis without clinically significant portal hypertension and an adequate functional liver remnant.
- Regarding transplant, criteria and exception points vary by province and by centre. Standardized national transplant criteria are needed.
- Vascular invasion and extrahepatic disease are general exclusion criteria while total tumour volume (TTV) and alpha-fetoprotein (AFP) criteria may vary by centre.

### 2.2. Summary of Evidence

Curative surgical resection is recommended in *non-cirrhotic patients* when there is an ability to achieve negative margins with adequate functional liver remnant. These patients have low post-operative liver morbidity, lower cumulative HCC recurrence rates, and higher disease-specific survival [1,2]. Metabolic dysfunction-associated fatty liver disease (MAFLD) patients have higher rates of peri-operative complications but improved disease-free and overall survival [1,2].

Selective resection is recommended in *cirrhotic patients* (i.e., a single lesion in a patient with compensated cirrhosis without clinically significant portal hypertension, and an adequate functional liver remnant) [3,4,5,6]. There may be exceptions to these criteria in select cases where transplant is not an option. Patients should be reassessed for transplant eligibility throughout their treatment journey.

Both *criteria and exception points vary by transplant centre*. We should work towards universal criteria within a universal healthcare system. In general, there is some variation of TTV and AFP criteria in Western Canada with vascular invasion and extra-hepatic disease being contraindications. Multidisciplinary management is recommended for both *down-staging and bridging* to transplant [7,8]. Locoregional ablation and transarterial radioembolization (TARE) are ideal, with transarterial chemoembolization (TACE) and immune checkpoint inhibitors (ICIs) acceptable [7,8]. ICIs should be discontinued 3 months prior to transplant to avoid increased risk of rejection and graft [7,8].

## 3. Question 2—What Are the Current Indications and Modalities for Non-Surgical Liver-Directed Therapies in the Treatment of Hepatocellular Carcinoma?

### 3.1. Recommendations

- All patients should be discussed at multidisciplinary team rounds.
- For tumours ≤ 3 cm in size, the modality option depends upon the local expertise, which may include radiofrequency ablation (RFA), microwave ablation (MWA), and stereotactic body radiation therapy (SBRT).
- For intermediate risk disease, TARE or stereotactic ablative body radiotherapy (SABR) is preferred subject to availability. If TARE or SABR are not available, then TACE is an alternative option.

### 3.2. Summary of Evidence—An Interventional Radiology Perspective

HCC is a common cancer and results in the third most cancer-related deaths [9,10]. Treatment is often aimed at optimizing patients for transplant due to the high risk of recurrence [11]. Liver resection is the gold standard for treatment of HCC in surgical candidates; however, many patients are not surgical candidates due to portal hypertension and comorbidities. As such, interventional radiology providing ablative and transarterial treatments is suitable for a large patient population. Ablation through RFA and MWA have been shown to be non-inferior to surgical outcomes in BCLC-0 (very early stage of HCC) and BCLC-A (early stage) patients [12,13,14]. The BCLC-B (intermediate stage) patient population is extremely heterogenous. In patients who are not transplant candidates, TARE and TACE are suitable locoregional treatment options [11,15]. Recent data has shown superior outcomes in appropriately dosed TARE-treated patients with increased overall survival and time to progression and improved downstaging [16]. We now have tools (i.e., TARE) which can facilitate downstaging and lead to liver transplant.

### 3.3. Summary of Evidence—A Radiation Oncology Perspective

#### 3.3.1. Introduction

Primary liver cancer cases include HCC, 75–85%, and intrahepatic cholangiocarcinoma (ICC), 10–15% [17,18]. Surgery including liver transplantation is the definitive treatment for HCC. Most primary HCC cases are not surgical candidates, however, and only about 20% present with resectable disease. Early-stage patients who undergo surgical resection have a 5-year survival rate of 50–70%, while patients with advanced disease have a 5-year recurrence rate of 70% or greater [17,18]. Multiple treatment modalities—including TACE, chemotherapy, and molecular targeted agents—are used to try to improve the survival rates in non-surgical cases [17]. This section will focus on radiotherapy as emerging experience and clinical trials show increasing promise in the treatment of intermediate and advanced HCC cases [17].

Radiotherapy is focused ionizing radiation and can be delivered as a non-invasive treatment from an external source. A number of techniques deliver external radiotherapy precisely, including SBRT, intensity-modulated radiation therapy (IMRT), volumetric-modulated arc therapy (VMAT), and three-dimensional conformal radiation therapy (3D-CRT). Alternatively, it can be delivered internally as brachytherapy (intra-luminal or interstitial), through a variety of techniques such as yttrium-90 microspheres, iodine-131 implantations, monoclonal antibodies, or iodinated oil [17].

Ionizing radiation causes direct and indirect DNA double-strand breaks. Where malignant cells have poor repair mechanisms, accumulated DNA damage causes cell death [18]. Liver cells and liver tumours are sensitive to radiation treatment. A normal liver has a strong ability to regenerate and spared normal liver tissue can compensate for radiation-induced focal damage through hepatic cell proliferation. Prior to the 1990s, however, the delivery of radiotherapy was less precise due primarily to limitations of the technologies available for imaging and precision focusing of the treatment beams. As a result, large-volume irradiation led to radiation-induced liver disease (RILD), liver failure, and sometimes death, which delayed the use of focal radiation for HCC. This experience continues to delay the adaptation of the newer radiotherapy treatment techniques for managing advanced cases of HCC [17,18,19].

#### 3.3.2. Focal Radiotherapy

Currently, 3D-CRT, IMRT, VMAT, and SBRT techniques are all contributing to increasing conformity in targeting disease more effectively, sparing normal tissues (including normal liver) and reducing the side effects of radiotherapy [17,18,19,20,21,22,23]. Treatment imaging and motion management techniques (image-guided radiation therapy (IGRT)) have also markedly improved increasing confidence in the accuracy of treatments [17,18,19,20,21,22,23].

Internal radiation therapy (brachytherapy) delivered percutaneously or intra-luminally has also evolved with the development of yttrium-90 microspheres, iodine-131 monoclonal antibodies, and iodine-125 particle implantation [17].

#### 3.3.3. 3D-CRT

The recent developments in 3D-CRT allow for more accurate and conformal treatment, while reducing the risk of RILD and other normal tissue toxicities. Local control of early-stage liver cancers can be as high as 73–81% at one year with single tumours (<10cm), although larger tumours have been successfully treated with this technique [17]. It has also been shown to be effective in cases with portal vein and in cases of inferior vena cava tumour invasion with local control rates up to 60% [17].

#### 3.3.4. IMRT

This technique varies the intensity of the radiation within the treatment fields to deliver a more uniform dose to the target tumour, while minimizing radiation to closely associated normal tissues. Clinical studies have mainly focused on advanced HCC cases, with local control ranging from 30 to 50% [17].

IMRT post-operative treatment for selected cases with narrow resection margins has been shown to achieve overall survival and relapse-free survival similar to wide-resection-margin cases. While VMAT is a newer and more dynamic form of the IMRT method, it improves conformity to the tumour target, and dose uniformity in the treatment area [17].

#### 3.3.5. SBRT

With image guidance and respiratory motion management technology, SBRT can be used to deliver very high doses to very conformal targets. There is very rapid dose fall-off at the treatment edges, which helps to reduce dose to closely associated normal tissues. This allows radiation to be delivered in fewer treatments with higher doses delivered to malignant cells per treatment. Current treatment regimens consist of 1 to 6 treatments over 1 to 2 weeks, versus standard fractionations of 25 to 35 treatments over 5 to 7 weeks [17,18,19,20,21,22,23].

##### SBRT in HCC

Most prospective trials have been phase II trials looking at definitive SBRT treatment for HCC. Trials varied in tumour size treated (ranging from up to 10 cm in one trial, and up to 23 cm in another) [17,21]. Despite this variability the two-year local control rates across trials range from 82% to 100% [21]. The overall survival (OS) at 2 years has ranged from 68% to 96% [21,23]. One trial had OS for Child-Pugh A (CP-A) at 72% and Child-Pugh B (CP-B) at 32.7% [20].

Across trials, SBRT dose has varied from R30 to 60 Gy in three fractions, or 35–40 Gy in five fractions, with reduced dose for CP-B cases in one trial [17,20,21]. The toxicities have also ranged widely with Grade 3+ toxicities ranging from 2.8% to 31% [18]. CP-B also had grade 3+ toxicity 38% [20]. Variation in toxicities may relate to base liver function, tumour size, and definition of treatment-related toxicities [17,18,19,20,21,22,23].

One review of studies found most success has been reported with treatment of smaller liver cancers [18]. They found across studies that SBRT showed 3-year local control of 68–97%, and 3-year survival of 39–84% [18]. The variability in survival is attributed mainly to underlying liver disease [18].

SBRT also has some treatment overlap with radiofrequency ablation. Comparison trials have not shown a definitive advantage for either treatment modality for lesions of 2 cm or less [18,21].

##### SBRT in Advance HCC

Multiple review studies show local control of 63–87% in patients with large, more advanced HCC often invading the portal vein, with risk of liver toxicity ranging from 10–30% [19,23]. Median survival rates are reported from 12.9 to 17 months [23]. 

Reviews of retrospective studies show comparable tumour control between thermal ablation and SBRT (80.2% vs. 83.8%) [19,22], with improved control rates for SBRT in tumours greater than 2 cm [19].

Regarding SBRT versus TACE for primary tumour control (91% vs. 23% at 2 years) [22], SBRT is comparable to other treatments when bridging to transplant [18,23].

#### 3.3.6. Conclusions

External beam radiotherapy has evolved, and preliminary phase II studies are showing that radiotherapy does have a role in the management of HCC. Delivery of external beam radiotherapy is a non-invasive procedure that is highly adaptable to particular clinical cases. Although potentially cost-effective and readily available, this treatment modality requires more economic analyses to be included in studies [24,25].

Provincial cancer care organizations should be encouraged to develop expertise for delivery of radiotherapy/SBRT for HCC. Opening and enrolling patients into multicentre randomized controlled trials not only helps answer clinical questions around radiotherapy for HCC, but also helps centres develop the technical skills to deliver these treatments safely and accurately. One example of the work being done is the SOCRATES HCC trial, a randomized controlled trial of standard of care versus radiofrequency ablation in early-stage HCC [26]. This phase II study is comparing SABR to current standard of care therapies focused on solitary HCCs ≤ 8 cm that are ineligible for surgical resection or transplant. Cohort 1 includes tumours ≤ 3 cm and evaluates thermal ablation versus SABR. Cohort 2 includes tumours > 3 cm to 8 cm and evaluates SABR versus best other standard of care therapy including transarterial therapies. The primary outcome for both cohorts is freedom from local progression at 2 years. The secondary outcomes are progression-free survival (PFS), OS, adverse events, patient reported outcomes, and a health economic analysis [26].

## 4. Question 3—What Is the Optimal First-Line Systemic Treatment for HCC and Is There a Role for Combining Locoregional and Systemic Therapy?

### 4.1. Recommendations

- Combination therapy with bevacizumab + atezolizumab, or combination immunotherapy are preferred first-line options in patients with newly diagnosed advanced CP-A score HCC.
- First-line lenvatinib (preferred) or sorafenib can be considered in patients with a contraindication to immunotherapy. For select CP-B-7 patients, lenvatinib or durvalumab + tremelimumab (Durva/Treme) can be considered.
- A combination of local and systemic therapy in intermediate-risk HCC have shown promising results; however, mature data is required before incorporating this therapy into clinical practice.

### 4.2. Summary of Evidence

First-line systemic therapy has evolved in recent years. Prior to the development of combination therapy, the standard of care for patients with advanced HCC CP-A was either sorafenib or lenvatinib. Sorafenib was found to be superior to placebo in the SHARP trial (hazard ratio [HR] in the sorafenib group, 0.69; 95% confidence interval (CI), 0.55–0.87; *p* < 0.001) and the ASIA-PACIFIC trial (HR 0.68, 95% CI, 0.50–0.93; *p* = 0.014) [27,28]. In the REFLECT trial lenvatinib was non-inferior to sorafenib in overall survival in untreated advanced HCC [29]. Based on the more favourable toxicity profile of lenvatinib, it became the favoured targeted therapy.

The IMBrave150 trial compared the combination of atezolizumab (1200 q3 weeks) + bevacizumab (15 mg/kg 3 weeks) versus sorafenib, in patients with CP-A advanced and metastatic HCC [30]. Patients were evaluated for any oesophageal or gastric varices, which were required to be treated according to local clinical practice prior to enrolment in the trial [30]. The combination was found to be superior in both the primary endpoints of progression-free survival (0.59; 0.47–0.76; *p* < 0.001) and overall survival (HR 0.58; 0.42–79; *p* < 0.001 [30]. Toxicity was manageable and based on these results this treatment regimen became the preferred therapy for patients with CP-A HCC.

The HIMALAYA trial evaluated the immunotherapy regimen of tremelimumab (300 mg × 1 dose) + durvalumab (1500 mg q 4 weeks; STRIDE) versus durvalumab (1500 mg every 4 weeks versus sorafenib (400 mg twice daily) [31]. The primary endpoint of overall survival of the STRIDE regimen versus sorafenib was achieved 0.78 (96.02% CI, 0.65–0.93; *p* = 0.0035) [31]. Toxicity with grade 3/4 treatment-related adverse events was experienced in 25.8% of patients on the STRIDE regimen [31].

In the Checkmate-9DW trial presented at the 2024 American Society of Clinical Oncology (ASCO) annual meeting, first-line nivolumab (1 mg/kg) + ipilimumab (3 mg/kg) (every 3 weeks; up to 4 cycles) followed by nivolumab 480 mg (every four weeks for up to two years) was shown to be superior to either sorafenib or lenvatinib (HR, 0.79; 95% CI 0.65–0.96; *p* = 0.018) [32]. This translated into a median overall survival of 23.7 (18.8–29.4 months [32]. There were no unexpected safety issues identified with this combination [32].

There is now accumulating evidence supporting the combination of local and systemic therapy in intermediate-risk HCC. The EMERALD-1 study compared durvalumab + bevacizumab + TACE versus TACE + placebo in CP-A-B7 patients [33]. The progression-free survival was superior in the experimental arm (HR 0.77; 95% CI 0.61–0.98; *p* = 0.032) [33]. The LEAP-012 trial evaluated lenvatinib plus pembrolizumab in combination with TACE versus TACE alone [34]. At the first interim analysis the combination significantly improved progression-free survival compared with TACE alone (HR 0.66; 95% CI 0.51–0.84; *p* = 0.0002) [34].

## 5. Question 4—What Are the Key Molecular Tests in Biliary Tract Cancer to Identify Actionable Mutations for Targeted Therapy?

### 5.1. Recommendations

- Patients need to be counselled prior to molecular testing, as mutations may not be actionable, or if drug access is limited. If targeted treatment is accessible, then the following tests can impact treatment decisions.
- Potential actionable mutations include IDH-1, HER 2 amplification, BRAF V600E, NTRK fusion gene, and RAS.
- DNA mismatch repair (MMR) testing is recommended for all patients.
- Other investigational markers may be used for clinical trial eligibility.

### 5.2. Summary of Evidence

Biliary tract cancer, which includes cholangiocarcinoma (CCA) and gallbladder adenocarcinoma, is a rare but aggressive malignancy with poor prognosis. The biliary tree is subdivided into small and large intrahepatic ducts where the small ducts are lined by cuboidal cholangiocytes and the larger ones are lined by both columnar and mucoid cholangiocytes. The extrahepatic biliary tree shares the anatomy of large ducts. Conventional intrahepatic cholangiocarcinoma has two variants—a small duct sub-type arising from smaller biliary ducts and a large duct type cholangiocarcinoma involving larger ducts [35]. The small duct sub-type is commonly seen within the peripheral hepatic parenchyma and immunohistochemical stains such as CD56 or N-Cadherin may be useful in detecting this origin. The large duct sub-type commonly occurs proximal to the hilum and can infiltrate the periductal region. Biliary intra-epithelial neoplasia and intra-tubular papillary neoplasm are some of the precursor lesions and immunohistochemical stains such as MUC5AC, MUC6, or S100P may be useful in identifying this sub-type. Risk factors for gallbladder adenocarcinoma include (but are not limited to) chronic cholelithiasis/cholecystitis and metaplasia.

The process of carcinogenesis, tumour evolution, and growth involves complex and heterogeneous processes that include the interplay of extracellular ligands (such as pro-inflammatory cytokines, growth factors, and bile acids, among others), which are present in the tumour microenvironment, and increased expression and/or aberrant activation of cell surface receptors and the deregulation of intracellular signalling pathways, finally leading to cell proliferation, survival, and migration or invasion. The most common genes that might be mutated or amplified resulting in the overactivation of some of these pathways include KRAS, BRAF, ARID1, PBRM1, BAP1, IDH1, and IDH2 [35].

The molecular landscape of biliary tract cancer is complex, and molecular profiling has become increasingly important for understanding the underlying biology of the disease and identifying actionable mutations for targeted therapies. Here is an overview of the more common molecular profiling and actionable mutations in biliary tract cancer:

- Isocitrate dehydrogenases 1 and 2 (IDH1/2) mutation [35,36,37]

These mutations are present more in iCCA, particularly in early-stage tumours. Mutant IDH1 (R132H) leads to the accumulation of 2-hydroxyglutarate (2-HG), which is associated with epigenetic dysregulation. Both IDH1/2 lead to the production of increased levels of 2-HG, which interferes with histone and DNA demethylases and leads to inhibition of the mitochondrial electron transport chain (epigenetic dysregulation).

- KRAS mutation

Observed in just over a third of extrahepatic cholangiocarcinomas and some gallbladder carcinoma, these mutations often lead to activation of the mitogen-activated protein kinase (MAPK) signalling pathway, contributing to tumorigenesis.

- BRAF mutation [38]

Found more commonly in intrahepatic cholangiocarcinoma (BRAF V600E), these mutations can lead to constitutive activation of the MAPK/extracellular-signal-regulated kinase (ERK) signalling pathway, which regulates cell differentiation.

- Tumour protein p53 (TP53) mutation [35]

One of the most prevalent mutations at approximately 47%, these mutations are more commonly seen in extrahepatic cholangiocarcinoma and gallbladder cancer as opposed to intrahepatic cholangiocarcinoma. TP53 mutations are linked to genomic instability and poorer prognosis as compared with patients with IDH1/2 mutations.

- Human epidermal growth factor receptor 2 (HER2) mutation [39]

Prevalent in 5–15% of the carcinomas (on average), it can be easily interpreted using immunohistochemical and in situ hybridization techniques, making this one of the more accessible mutations for testing, along with MMR.

- MMR mutation [40]

Loss of expression has been linked to better prognosis due to improved response to immune checkpoint inhibitors. Immunohistochemical testing with loss in the expression of one or more of the proteins (MLH1/PMS2 or MSH2/MSH6) is considered MMR deficient.

- Programmed death ligand 1 (PD-L1) [41]

Expressed as a potential predictive biomarker, this mutation is prevalent in approximately 7–9% of tumours. It is assessed using immunohistochemical testing and a Combined Positive Score (CPS) scoring system. CPS ≥ 5% is indicative of positive PD-L1 expression.

## 6. Question 5—What Is the Role of Adjuvant Radiation Therapy for Patients with R1 Resections in Biliary Tract Cancer and How Should It Be Incorporated with Adjuvant Systemic Therapy?

### 6.1. Recommendations

- Evidence for adjuvant radiation therapy (ART) in this setting is based on small single-arm or phase II studies and is not strong. ART is not routinely recommended in this setting. If ART is to be administered, then priority should be given to administering systemic therapy first followed by ART (either alone or adjuvant chemoradiation (CRT)).
- All patients should be discussed at multidisciplinary team rounds.
- Patients should be considered for clinical trials if available.

### 6.2. Summary of Evidence

#### 6.2.1. Introduction

Biliary tract cancers (BTCs) are rare but aggressive malignancies with poor prognoses. Surgical resection remains the cornerstone of curative treatment; however, high locoregional recurrence rates necessitate adjuvant strategies. This review examines the current evidence supporting ART in BTC, with a focus on the published literature and an elaborate discussion of findings specific to intrahepatic cholangiocarcinoma (ICC), extrahepatic cholangiocarcinoma (ECC), and gallbladder cancer (GBC).

BTC encompasses a heterogeneous group of malignancies, including ICC, ECC, and GBC. Despite advances in surgical and systemic therapies, outcomes remain suboptimal due to high recurrence rates. ART has been proposed to improve locoregional control and overall survival. This review synthesizes data from clinical trials, retrospective analyses, and meta-analyses to evaluate the role of ART in BTC management.

#### 6.2.2. Rationale for Adjuvant Radiation Therapy

Surgical resection offers the best chance for cure in BTC. However, complete resection (R0) is often challenging due to anatomical constraints and the proximity of critical structures. Even after R0 resection, recurrence rates range from 50% to 70%, with locoregional failure accounting for a significant proportion [42,43]. This underscores the potential role of ART in mitigating recurrence. The rationale for ART in biliary tract cancers lies in its ability to target microscopic residual disease and high-risk locoregional areas. ART aims to improve locoregional control by delivering high-dose radiation to the tumour bed and regional lymph nodes, reducing the likelihood of residual tumour cell proliferation. While systemic metastases remain a concern, ART’s ability to control locoregional disease contributes significantly to long-term survival outcomes. ART is particularly beneficial in patients with R1 resections, lymphovascular invasion, or positive lymph nodes with the highest risk of recurrence. Modern radiation therapy techniques have significantly improved the precision and efficacy of ART in biliary tract cancers. Intensity-modulated radiation therapy (IMRT) allows for highly conformal dose distributions, sparing adjacent normal tissues such as the liver, kidneys, and gastrointestinal structures. Proton beam therapy offers dosimetric advantages by minimizing radiation exposure to non-target tissues, reducing toxicity, and enabling dose escalation [44]. Image-guided radiation therapy (IGRT) enhances accuracy by accounting for daily variations in patient anatomy, ensuring precise targeting of the tumour bed. Adaptive radiation therapy enables real-time modifications to treatment plans based on changes in tumour size or patient anatomy during therapy.

#### 6.2.3. Evidence Supporting Adjuvant Radiation Therapy

##### Intrahepatic Cholangiocarcinoma (ICC)

Intrahepatic cholangiocarcinoma (ICC) has distinct biological behaviour, with a high risk of intrahepatic and distant metastases. The role of ART remains controversial but may benefit high-risk patients. A retrospective study by Kim et al. showed that ART reduced locoregional recurrence rates in ICC patients with R1 resection or nodal involvement, although the overall survival benefit was limited [42]. Similarly, a study by Wang et al. reported improved locoregional control with ART in high-risk ICC patients [45]. Advances in techniques such as proton beam therapy and IMRT have demonstrated the potential to improve locoregional control while reducing toxicity. Kim et al. highlighted the dosimetric advantages of proton therapy, which minimizes damage to healthy liver tissue [42]. Additionally, a meta-analysis by Ke et al. found that ART improved locoregional control in ICC, particularly in patients with positive margins or lymph node involvement [46].

##### Extrahepatic Cholangiocarcinoma (ECC)

Extrahepatic cholangiocarcinoma (ECC) has a high propensity for locoregional recurrence. Ren et al. reported improved overall survival and locoregional control with ART, particularly in patients with positive surgical margins or nodal positivity [47]. The SWOG S0809 trial demonstrated that adjuvant chemoradiotherapy resulted in a 2-year survival rate of 65% (95% CI 53%–74%) [48]. A retrospective analysis by Zhou et al. confirmed the benefit of ART in reducing recurrence rates in ECC patients with high-risk features [49]. Institutional studies, such as that by Ali et al., observed improved 5-year survival in ECC patients receiving ART compared with surgery alone, reinforcing its role in high-risk patients [50]. Mechanistically, ART targets residual microscopic disease and perineural invasion, which are common in ECC. The Mayo Clinic experience underscored its efficacy in improving outcomes in patients with R1 resections [51].

##### Gallbladder Cancer (GBC)

Gallbladder cancer (GBC) is often diagnosed at advanced stages, with a high likelihood of nodal involvement and peritoneal spread. This makes ART an attractive option for reducing recurrence and improving survival in select patients. Hoehn et al. analysed data from the National Cancer Database, showing improved OS in GBC patients with R1 resection margins or nodal positivity who received ART [52]. A retrospective study by Wang et al. demonstrated a model that predicted certain subsets of patients with at least T2 or N1 disease will gain a survival benefit from adjuvant CRT, and the magnitude of benefit for an individual patient can vary [53]. Systematic reviews by Horgan et al. and Ren et al. confirmed ART’s role in reducing locoregional recurrence and improving overall survival in high-risk GBC patients [47,54]. Emerging techniques such as IMRT and image-guided radiation therapy (IGRT) have enhanced the precision of ART, enabling effective targeting of high-risk areas while sparing adjacent organs.

##### Clinical Guidelines and Recommendations

Current clinical guidelines recommend considering ART in high-risk biliary tract cancer patients, particularly those with positive or close surgical margins (R1/R2 resection), lymph node involvement, or perineural and lymphovascular invasion. The decision to pursue ART should be made within a multidisciplinary team, considering patient performance status, comorbidities, and preferences.

#### 6.2.4. Challenges and Future Directions

Despite promising data several challenges remain. The heterogeneity of biliary tract cancers, with variability in tumour biology and anatomical location, complicates the standardization of ART protocols. ART can lead to gastrointestinal and hepatobiliary toxicity, necessitating careful patient selection and treatment planning. The paucity of randomized trials limits definitive conclusions about ART’s efficacy. Future research should focus on prospective randomized trials to define the role of ART, integration of ART with novel systemic therapies, including immunotherapy and targeted agents, and biomarker-driven approaches to identify patients most likely to benefit from ART.

#### 6.2.5. Conclusions

Adjuvant radiation therapy plays a critical role in the multidisciplinary management of biliary tract cancers, particularly in patients with high-risk features such as R1 resection and nodal positivity. Advances in radiation techniques and emerging systemic therapies hold promise for improving outcomes. Further research is needed to establish standardized protocols and optimize patient selection.

## 7. Question 6—What Are the Optimal First- and Second-Line Systemic Treatments for Advanced Biliary Tract Cancer with or Without Molecular Profiling Information?

### 7.1. Recommendations

- Clinical trial participation should be encouraged when available.
- A combination of platinum, gemcitabine, and immunotherapy with durvalumab or pembrolizumab is the optimal systemic treatment, if available.
- The benefits of second-line therapy are limited, but fluoropyrimidine-based chemotherapy is preferred and could be combined with oxaliplatin or irinotecan-based treatment.
- Molecular testing does not alter first-line treatment options outside of a clinical trial.
- In the second-line setting, if targeted therapy is available, then it would be preferred over conventional second-line chemotherapy. Best supportive care is always an option.

### 7.2. Summary of Evidence

A combination of cisplatin, gemcitabine and immunotherapy with durvalumab or pembrolizumab is the optimal systemic treatment in the first-line setting. The chemotherapy backbone of cisplatin and gemcitabine was established as a standard of care by the ABC-02 trial which demonstrated a median overall survival of 11.7 from 8.1 months when compared with gemcitabine alone (HR 0.64; 95% CI 0.52–0.80; *p* < 0.001) [55]. Two immunotherapy drugs established similar interval improvements in survival when added to this chemotherapy backbone, with durvalumab in the TOPAZ-1 study and pembrolizumab in the KEYNOTE-966 trial, leading to median overall survival of 12.9 (HR 0.76, 95% CI 0.64–0.91) and 12.7 months (HR 0.83, 95% CI 0.72–0.95; *p* = 0.0034), respectively [56,57]. In the absence of a contraindication to immunotherapy, either combination can be considered as first-line treatment.

In the absence of a targetable mutation, the best evidence for second-line treatments comes from the 162-patient phase III clinical trial ABC-06 [58]. This trial demonstrated a modest, but statistically significant improvement in median overall survival from the combination of fluoropyrimidine 5-FU + oxaliplatin (FOLFOX) plus active symptom control (ASC) compared with ASC alone, from 5.3 to 6.2 months (HR 0.69, 95% CI 0.50–0.97; *p* = 0.031) [58]. However, the use of oxaliplatin—particularly with its increased toxicities such as neuropathy—may not have a clear incremental benefit beyond fluoropyrimidine alone. A 321-patient retrospective analysis in this setting suggests that there is no clear incremental improvement with a fluoropyrimidine alone versus a combination of fluoropyrimidine and a platinum agent such as oxaliplatin, with a median overall survival of 6.5 vs. 6.2 months, *p* = 0.88 [59]. The NIFTY phase II clinical trial evaluated the impact of nano-liposomal irinotecan with a fluoropyrimidine compared with a fluoropyrimidine alone, and demonstrated an improved median progression-free survival of 7.1 vs. 1.4 months (HR 0.56, 95% CI 0.39–0.81; *p* = 0.0019) as its primary outcome, and a median overall survival of 8.6 versus 5.3 months (HR 0.68, 95% CI 0.48–0.95; *p* = 0.02), respectively [60]. Nano-liposomal irinotecan is not currently funded for this indication in Canada, and the fact that this is only a phase II trial limits the adoption of this approach. It is unknown if conventional irinotecan—which may be easier to access in Canada—would have a similar benefit beyond a fluoropyrimidine alone. Therefore, fluoropyrimidine-based treatments can be considered in the second-line setting for a modest benefit, while the incremental benefit of oxaliplatin or an irinotecan-based combination must have the risks and benefits weighed.

Cholangiocarcinomas are relatively rare, and when grouped together with other cancers of the biliary tract and liver, they only represent the 14th most common cancer in Canada [61]. Despite how uncommon they may be, they are more likely to harbour potentially actionable mutations than many other solid-organ malignancies, and they are one of the cancers for which next-generation sequencing is routinely recommended by ESMO [62]. Those with potentially actionable mutations represent unique subtypes and are challenging to study due to the overall small number of patients with the disease, so patients should be encouraged to participate in clinical trials whenever possible. Due to the rarity of these subtypes, the existing data to support targeted treatments have mainly consisted of phase I and II data in the second-line setting or beyond. Coupled with the high mortality rate of this disease, as the 6th most common cause of cancer-associated death for both males and females, any advances in treatment would fill an unmet need. The Cholangiocarcinoma Collaborative (C3) is a charitable organization that seeks to translate hope into better outcomes for patients facing cholangiocarcinoma. It offers resources for patients, caregivers, and healthcare providers including the facilitation of molecular testing in Canada and clinical trial navigation.

Approximately 10–15% of patients with an intrahepatic cholangiocarcinoma harbour a fibroblast growth factor receptor (FGFR2) fusion or rearrangement and may benefit from an FGFR2 inhibitor in the second-line setting. Pemigatinib demonstrated an objective response rate of 35.5% [95% CI 26.5–45.4] compared with 0% for patients with an alternate FGFR alteration or no alteration in the phase 2, open-label FIGHT-202 trial [63]. This study also demonstrated a median progression-free survival of 6.9 months (95% CI 6.2–9.6) and median overall survival of 21.1 months [95% CI 14.8 to not estimable]. Pemigatinib is accessible in Quebec and Alberta for patients with the appropriate mutation, and a broader provincial funding mechanism may be revisited. Other FGFR2 inhibitors that have shown responses include derazantinib, erdafitinib, futibatinib, infigratinib, and lirafugratinib (RLY-4008), but none are readily accessible for this indication in Canada outside of a clinical trial [64,65,66,67,68]. Infigratinib initially received Health Canada approval for this indication and had received accelerated approval by the FDA, but this was ultimately withdrawn due a lack of further data.

An isocitrate dehydrogenase 1 (IDH1) mutation is present in approximately 20% of intrahepatic cholangiocarcinomas [36]. The ClarIDHy trial demonstrated a statistically significant improvement in progression-free survival from 1.4 to 2.7 months (HR 0.37, 95% CI 0.25–0.54; *p* < 0.0001) with the use of ivosidinib versus placebo [69]. Patients who received placebo were allowed to cross over to ivosidinib and the median overall survival of 10.3 compared with 7.5 months (HR 0.79, 95% CI 0.56–1.12; *p* = 0.09), which was not statistically significant [70]. Ivosidenib is Health Canada approved but is not specifically funded or accessible in Canada for cholangiocarcinoma treatment outside of a clinical trial.

Neurotrophic tyrosine receptor kinase (NTRK) fusion mutations are rare, but when they are present, objective responses have been demonstrated with the NTRK fusion inhibitors larotrectinib (1 of 2 patients) and entrectinib (1 of 1 patient) in clinical trials [71,72]. Both drugs are accessible in Canada, though longer term outcomes specific to cholangiocarcinomas are unknown.

Human epidermal growth factor receptor 2 (Her-2/ERB2) amplifications may be sensitive to targeted therapies, with responses demonstrated in trials with trastuzumab and pertuzumab, trastuzumab and tucatinib, trastuzumab-deruxtecan, and other agents [73,74,75,76,77,78]. Although trastuzumab-deruxtecan demonstrated an objective response rate of 36.4% in HER-2 positive disease in a phase II trial, two patients (25%) had a fatal interstitial lung disease [77]. KRAS G12C mutations may stabilize or respond to targeted inhibitors like sotorasib and adagrasib [79,80]. BRAF V600E mutations may respond to combined BRAF/MEK inhibition with agents like dabrafenib/trametinib [81]. None of these treatments are funded for this specific indication, but they may be accessible as part of a clinical trial.

## Figures and Tables

**Table 1:** Attendees at the 26th Annual WCGCCC.

Name	Specialty	Organization
Shahid Ahmed	Medical Oncologist	Saskatchewan Cancer Agency
Blaire Anderson	Hepatobiliary/Transplant Surgeon	University of Alberta
Brady Anderson	Medical Oncologist	Western Manitoba Cancer Centre
Bryan Brunet	Radiation Oncologist	Saskatchewan Cancer Agency
Haji Chalchal	Medical Oncologist	Allan Blair Cancer Centre
Arun Elangovan	Radiation Oncologist	Saskatoon Cancer Centre
Georgia Geller	Medical Oncologist	BC Cancer Victoria
Vallerie Gordon	Medical Oncologist	CancerCare Manitoba
Branawan Gowrishankar	Medical Oncologist	Cross Cancer Institute
Edward Hardy	Medical Oncologist	BC Cancer/IHA
Mussawar Iqbal	Medical Oncologist	Allan Blair Cancer Centre
Duc Le	Radiation Oncologist	Saskatoon Cancer Centre
Richard Lee-Ying	Medical Oncologist	Tom Baker Cancer Centre
Shazia Mahmood	Radiation Oncologist	Saskatchewan Cancer Agency
Karen Mulder	Medical Oncologist	Cross Cancer Institute
Maged Nashed	Radiation Oncologist	CancerCare Manitoba
Killian Newman	Interventional Radiologist	Foothills Medical Centre
Maurice Ogaick	Hepatobiliary Surgeon	University of Saskatchewan
Vibhay Pareek	Radiation Oncologist	CancerCare Manitoba
Jennifer Rauw	Medical Oncologist	Cross Cancer Institute
Deepti Ravi	GI/Liver Pathologist	Saskatchewan Health Authority
Ralph Wong	Medical Oncologist	CancerCare Manitoba
Adnan Zaidi	Medical Oncologist	Saskatoon Cancer Centre

**Table 2:** Clinical Questions Addressing Specific Aspects of Interest in Hepatocellular and Biliary Tract Cancers are Addressed as Part of the 26th Annual WCGCCC.

Clinical Questions (In Order of Discussion)	
1	What are the current criteria for curative surgery and transplant candidacy in patients with hepatocellular carcinoma (HCC)?
2	What are the current indications and modalities for non-surgical liver-directed therapies in the treatment of HCC?
3	What is the optimal first-line systemic treatment for HCC and is there a role for combining locoregional and systemic therapy?
4	What are the key molecular tests in biliary tract cancer to identify actionable mutations for targeted therapy?
5	What is the role of adjuvant radiation therapy for patients with R1 resections in biliary tract cancer and how should it be incorporated with adjuvant systemic therapy?
6	What are the optimal first- and second-line systemic treatments for advanced biliary tract cancer with or without molecular profiling information?
